# Neutrophil sub-types in maintaining immune homeostasis during steady state, infections and sterile inflammation

**DOI:** 10.1007/s00011-023-01737-9

**Published:** 2023-05-22

**Authors:** Kailash Ganesh, Manjunath B. Joshi

**Affiliations:** Department of Ageing Research, Manipal School of Life Sciences, Manipal Academy of Higher Education, Planetarium Complex, Manipal, 576104 India

**Keywords:** Neutrophils, Granulopoiesis, Heterogeneity, Auto immune diseases, Infections

## Abstract

**Introduction:**

Neutrophils are component of innate immune system and a) eliminate pathogens b) maintain immune homeostasis by regulating other immune cells and c) contribute to the resolution of inflammation. Neutrophil mediated inflammation has been described in pathogenesis of various diseases. This indicates neutrophils do not represent homogeneous population but perform multiple functions through confined subsets. Hence, in the present review we summarize various studies describing the heterogeneous nature of neutrophils and associated functions during steady state and pathological conditions.

**Methodology:**

We performed extensive literature review with key words ‘Neutrophil subpopulations’ ‘Neutrophil subsets’, Neutrophil and infections’, ‘Neutrophil and metabolic disorders’, ‘Neutrophil heterogeneity’ in PUBMED.

**Results:**

Neutrophil subtypes are characterized based on buoyancy, cell surface markers, localization and maturity. Recent advances in high throughput technologies indicate the existence of functionally diverse subsets of neutrophils in bone marrow, blood and tissues in both steady state and pathological conditions. Further, we found proportions of these subsets significantly vary in pathological conditions. Interestingly, stimulus specific activation of signalling pathways in neutrophils have been demonstrated.

**Conclusion:**

Neutrophil sub-populations differ among diseases and hence, mechanisms regulating formation, sustenance, proportions and functions of these sub-types vary between physiological and pathological conditions. Hence, mechanistic insights of neutrophil subsets in disease specific manner may facilitate development of neutrophil-targeted therapies.

## Introduction

Neutrophils are one of the important components of the innate immune system and play a crucial role in protecting the host against pathogens. Neutrophils express germline encoded pattern recognition receptors (PRRs) to recognize pathogens and lead to the activation of effector functions such as production of reactive oxygen species, degranulation, phagocytosis and formation of extracellular traps [1]. In response to circadian rhythms, anatomical location, infections, sterile inflammation and ageing, neutrophils adapt themselves to physiological or pathological roles and drive various biological functions [2–5]. Protozoans, fungi, bacteria and viruses stimulate neutrophils to expel their DNA along with granular proteins to form web like structures referred as extracellular traps (NETs) [6, 7]. High concentrations of antimicrobial effectors within these DNA lattices serve as a platform to activate pro-inflammatory mediators, immobilize and kill the pathogens, and simultaneously clear the infections. Aged and dead neutrophils along with accumulated NETs in inflamed tissues are cleared by macrophages and facilitate the resolution of inflammation [8]. Failure in neutrophil apoptosis and impaired clearance of dead neutrophils along with NETs components results in host tissue damage and release of pro-inflammatory cytokines [9–12]. Neutrophils also modulate functions of other cell types including vascular endothelial cells to induce pathological angiogenesis [13] and adaptive immune responses [14–17]. Accordingly, neutrophil dysfunction affects host pathophysiology and subsequently plays a role in the pathogenesis of diseases associated with sterile inflammation such as Type 2 diabetes (T2D), autoimmune diseases, vascular disorders, digestive disorders and a variety of malignancies [4, 10, 18–23].

Neutrophils were traditionally assumed to represent the homogenous population of terminally differentiated cells with definite functions. However, mounting evidence indicate the existence of neutrophil subtypes based on buoyancy, cell surface markers, localization and maturity [1]. Using pre-clinical and clinical models, studies have demonstrated functional relevance of neutrophil sub-types in both physiological conditions and pathologies associated sterile inflammation and infections. Khoyratty et al. employing RNAseq and chromatin profiling in neutrophils during their development, activation, tissue distribution and acute infection identified distinctly activated transcription factor networks indicating existence of neutrophil heterogeneity. Authors showed that KLF6 and RUNX1 were necessary for neutrophil maturation and survival; RELB, IRF5, and JUNB transcription factors were responsible for cytokine generation; ROS production and NETosis required RELB and JUNB, and phagocytosis was dependent on IRF5 and JUNB. [24]. Ballesteros et al. showed existence of tissue-specific neutrophil phenotypes based on single cell RNA and ATAC-seq analysis [25]. Neutrophil subtypes characterized based on buoyancy referred as low-density neutrophils (LDNs) have been demonstrated for their role in pathogenesis of various diseases including infections caused by HIV-1 and SARS-CoV-2, serum erythematous lupus and breast cancer [17, 26–28]. Accordingly, cell surface markers such as olfactomedin, CD177, CXCR4^+^CD62L^−^ and many others have also been basis of defining neutrophil subtypes which showed functional alterations in diseases [29–31]. Moreover, studies from many laboratories, including ours, have shown activation of stimulus-specific signalling pathways in neutrophils [32, 33]. For example, high glucose, LPS and homocysteine representing stimuli for diabetes, infections and thrombosis, respectively, induced distinct set of kinases which were associated with specific functions of neutrophils [33]. This indicates the existence of neutrophil subtypes with confined functions in patho(physiological) conditions and further, molecular mechanisms regulating the formation, sustenance and functions of these subsets also may vary among the diseases. Moreover, these tissue-to-tissue differences and their proportions may alter in pathological conditions. Hence, mechanistic insights of neutrophil subsets in disease-specific manner may facilitate development of neutrophil targeted therapies.

In the present review, we summarize various seminal studies describing the heterogeneous nature of neutrophils during steady-state and pathological conditions. Further, we discuss challenges and therapeutic opportunities for the management of neutrophil centered diseases.

## Neutrophil homeostasis during normal physiological conditions and infections

**Fig. 1:**
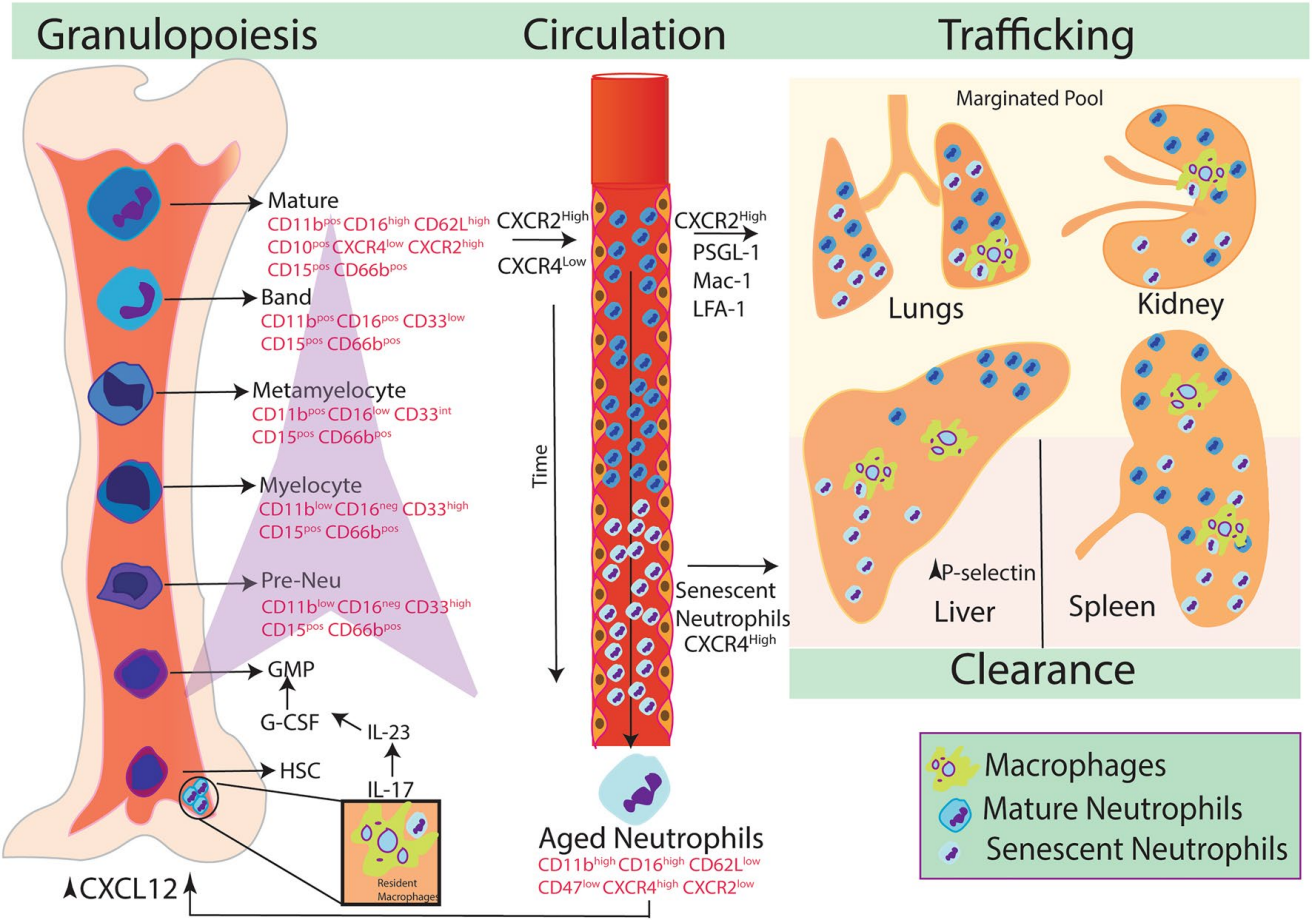
Life cycle of neutrophils in steady state. HSCs through series of differentiation forms promyelocyte, myelocyte, metamyelocyte, band cell and finally mature neutrophil. Steady increase in CXCR2 level and decrease in CXCR4 expression leads to release of neutrophils from the bone marrow to circulation. From circulation, neutrophils enter into various tissues majorly into lungs, liver and spleen. As neutrophils age, increase in the expression of CXCR4, migrates back into bone marrow for clearance by resident stromal macrophages. Neutrophils are also cleared in liver and spleen

Neutrophils develop from CD34 + hematopoietic stem cells and are terminally differentiated, small granulocytes with a remarkable short lifespan [34]. Neutrophil homeostasis is maintained at different phases of life cycle including production, trafficking and clearance (Fig. 1). Exogenous and endogenous factors that determine whether a hematopoietic stem cell (HSC) in bone marrow develops into a common lymphoid progenitor (CLP) or common myeloid progenitor (CMP) cell is not clearly understood [35]. While CLP precursors develop into either T cells, B cells or NK cells, CMP develops into a granulocyte monocyte progenitor (GMP) or a megakaryocyte erythroid progenitor (MEP) [36]. These GMPs commit to neutrophil formation by transitioning into myeloblasts under the control of granulocyte colony-stimulating factor (G-CSF). Subsequently, myeloblasts mature and pass through the phases of promyelocyte, myelocyte, metamyelocyte, band cell and finally to mature neutrophils (reviewed by Vietinghoff and Ley) [37]. The immature pools are generated by the differentiation of neutrophil precursor cells that differ from mature neutrophils in terms of morphology of nucleus, expression of granular proteins, proliferative potential and enhanced transcriptional activity [38]. Immature neutrophils in humans express CD15 and CD11b, followed by an increase in the expression of CD16 and CD10 as they mature [39]. In mouse, immature neutrophils are defined by the expression of CD11b^+^Ly6G^low^Ly6B^int^CD117^+^CD115^−^ and maturation begins with the elevated expression of Ly6G and decrease in the expression of progenitor cell marker c-Kit (CD117) [38]. Differential gene expression analysis in mature and immature neutrophils isolated from mouse bone marrow, blood and orthotopic pancreatic tumor showed elevated expression of *cd101*, which codes for a surface protein [40]. The increased expression of CD101 was observed in mature neutrophils in both bone marrow and peripheral blood. Based on the expression of CD101, authors characterized mature neutrophils with the expression of Ly6G^+^ CXCR2^+^ CD101^+^ and immature neutrophils as Ly6G^low^ CXCR2^−^ CD101^−^. Further, mouse with pancreatic tumors exhibited surge in immature neutrophil pool which might serve as a biomarker for disease progression [40]. Transcription factors such as CCAAT/enhancer binding proteins (C/EBPs), Runx1 and PU.1 regulate the path of neutrophil lineage [41–43]. The retention of neutrophils in the bone marrow depends on the expression of the chemokine CXCL12 by perivascular cells and osteoblasts, which is a ligand for the neutrophil cell membrane chemokine receptor CXCR4 [44]. As the neutrophils mature in bone marrow, the proportion of the chemokine CXCL2 and its cell membrane receptor CXCR2 expression increases while the levels of CXCR4 decrease. This leads to the release of neutrophils from the bone marrow to circulation [44].

Neutrophils are released from the bone marrow in a regulated fashion to maintain homeostatic levels and further, increase their number in response to stress, including infections. Circulating levels of neutrophils in physiological conditions are maintained by G-CSF [45]. Semerad et al. showed that nonredundant G-CSFR signals play an important role in regulating neutrophil release from the bone marrow and maintaining physiological levels of neutrophils in the blood [46]. However, neutrophil release during infections was independent of G-CSFR signalling [46]. G-CSF potently inhibits osteoblast activity resulting in decreased CXCL12 expression in the bone marrow and involved in regulation of the release of neutrophils into the blood stream [47]. ELR + chemokines such as CXCL1-CXCL3 and CXCL5-CXCL8 produced by bone marrow- endothelial cells and osteoblasts signals through CXCR1 and CXCR2 and opposes CXCR4/CXCL12 axis [44]. Eash et al. showed balance between these pathways directs neutrophils towards bone marrow vascular space for trafficking [44]. Sympathetic nervous system provides adrenaline signals to stromal cells through β3-adrenergic receptors in bone marrow to generate circadian rhythm and regulate the expression of *cxcl12* [48]. Circulating HSCs were elevated at 5 h after the initiation of day light and inversely correlated with the CXCL12 levels in BM and the release of neutrophils during the same period coincided with the levels of CXCL12 [48, 49]. Upon release into circulation, neutrophils also infiltrate abundantly into the lungs, spleen and bone marrow and however studies have demonstrated presence of neutrophils in the liver, intestine, white adipose tissue and skeletal muscles [45, 50].

Further under homeostatic conditions, aged neutrophils undergo clearance in different tissues. Aged neutrophils expressing decreased levels of CXCR2 and elevated CXCR4 expression leads to their migration towards bone marrow in response to CXCL12 (Fig. 1) [51]. Furze and Rankin using ^111^In-labelled aged neutrophils, demonstrated that ∼32% of neutrophils from the circulation were cleared in bone marrow in CXCR4 dependent manner and phagocytosed by resident stromal macrophages. Further, authors demonstrated approximately 29% and 26% of neutrophils were cleared in the spleen and liver, respectively [45]. Clearance of neutrophils in the liver was also observed in rat models of endotoxemia which was accompanied by an increase in P-selectin in hepatic sinusoids which led to phagocytosis of neutrophils by Kupffer cells [52].

Regulation of neutrophil production is classified into two types: steady state and emergency granulopoiesis. Changes in the microenvironment by external stimuli such as infections shift between these two stages. In steady-state granulopoiesis, ingestion of neutrophils by macrophages stimulates the activation of C/EBP-α in turn decreasing the production of cytokines and lowering the G-CSF level [53]. Whereas emergency granulopoiesis is associated with the excessive release of mature and immature neutrophils and concomitantly increase the proportion of immature neutrophils in circulation. Infections induce C/EBP-β expression and elevate cytokine levels of granulocyte–macrophage colony-stimulating factor (GM-CSF), G-CSF, Interleukin (IL) -1β and tumor necrosis factor (TNF) -α [54]. Interestingly, C/EBP- α and C/EBP- β both share common molecular interaction which are associated with granulopoiesis but differs only in the regulation of the cell cycle [55]. Emergency granulopoiesis is associated with elevated expression of chemokines such as macrophage inflammatory proteins (MIP), keratinocyte chemoattractant (KC), TNF-α and G-CSF that escalate the production of ROS in NADPH oxidase-dependent manner [54, 56]. Oxidation and deactivation of phosphate and tensin homologue (PTEN) in the myeloid cell are increased by ROS activity [57]. This leads to elevated production of G-CSF and activates emergency granulopoiesis [57].

## Heterogeneous nature of neutrophils during normal physiological state

Neutrophil subsets are observed in both physiological and pathological conditions [58]. Neutrophils exists in three different pools (a) proliferative, (b) circulating and (c) marginated (Fig. 1) and the percentage of each pool of cells is influenced by maturational development and an individual’s health [45]. RNA, protein and chromatin studies of mouse neutrophils from various anatomical regions including the bone marrow, gut, lung, spleen, skin and blood revealed heterogeneous properties of neutrophils [25, 59]. Balleteros et al., using single cell RNA sequence analysis demonstrated neutrophils acquired distinct transcriptional profiles and phenotype in tissue specific manner. Authors showed distinct transcriptional clusters of blood, lung, spleen and bone marrow infiltrated neutrophils whereas intestine and skin derived neutrophils clustered together. This indicated phenotypic heterogeneity of neutrophils across tissues in normal steady state [25]. On similar lines, Xie et al. identified five distinct clusters of neutrophils during developmental stage represented as G0, G1, G2, G3, G4 corresponding to BM-GMP, proNeu, preNeu, immature Neu and mature Neu, respectively. Authors also identified three transcriptionally distinct subpopulation of mature neutrophils in peripheral blood represented as G5a, G5b, and G5c where G5a expressed higher levels of *Mmp8* and *S100a8* responsible for neutrophil migration and inflammatory response [59]. G5b neutrophils expressed a set of interferon-stimulated genes such as Ifit3 and Isg15 may be primed to combat infections. Upon experimental bacterial infection, G0 and G1 showed elevated ROS levels indicating that these early progenitor cells were primed for immune adaptation. Also, G4 and G5 neutrophils displayed upregulation of genes responsible for secretion and cytokine production [59]. Dinh et al. using mass cytometry identified earliest neutrophil progenitor cells characterized by the expression of Lin^−^CD117^+^CD71^+^CD66b^+^ in human bone marrow neutrophil lineage [60]. Using single cell RNA sequencing methods, Wigerblad et al. identified four transcriptional clusters of neutrophils in human peripheral blood. Authors referred these clusters as Nh0 (transcriptome closely related to immature neutrophils); Nh1 (transitional phenotype); Nh2 (transcriptionally inactive) and Nh3 (enriched with transcripts related to type I IFN –inducible genes). These subsets were characterized by the expression of distinct set of transcription factors [61]. Evrard et al. identified committed proliferative neutrophil precursor (preNeu) in mice which differentiated into non-proliferating immature neutrophils and mature neutrophils. PreNeu cells ensured the production of neutrophils in homeostasis and stress [40]. Olsson et al. identified two intermediates of Gfi1-GMP positive populations (GG2, GG3) in Gfi1–GFP reporter mice. Among these two GG2 with high expression of Gfi1 represented granulocytic progenitors while GG3 expressed the highest level of Irf8 represented monocytic progenitor cells [62]. Using single cell RNA sequencing Huang et al. identified five distinct subgroups (G3, G4, G5a, G5b, G5c) of neutrophils in healthy and subjects with burnt wounds. These subsets were confined to distinct functions and showed significant alterations in transcriptome between healthy and burn conditions [63].

**Fig. 2:**
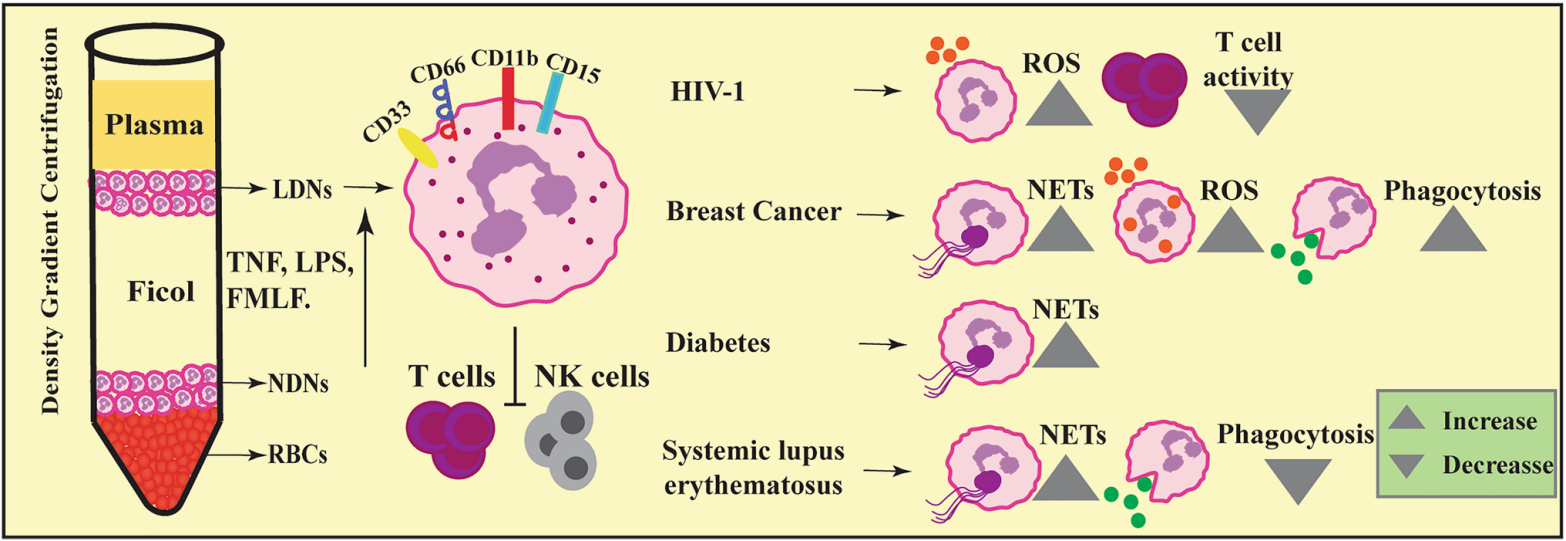
Low density neutrophils in diseases. Based on density neutrophils are characterized into low density neutrophils (LDNs) and normal density neutrophils (NDNs). LDNs are found in both normal physiology and pathological conditions. LDNs with surface marker CD33 CD66 CD11b CD15 found in several diseases such as HIV-1, cancer, diabetes and systemic lupus erythematosus which suppresses T cells and NK cells

Several studies have demonstrated neutrophil subtypes based on expression of cell surface proteins in both physiological and pathological conditions (Table 1). Under normal physiological state, proportion of neutrophils expressed NB-1 antigen (CD177) and upon stimulation of neutrophils with fMLP although NB-1 expression was increased and the quantity of NB-1 expressing cells did not alter [29]. Olfactomedin 4 (OLFM4), a glycoprotein was expressed only in 50% of human peripheral neutrophils however, both subsets of neutrophils with or without expression of OLFM4 participated in all neutrophil functions such as phagocytosis, degranulation, chemotaxis and NETs [30] (Fig. 4). B helper neutrophils residing in the perifollicular region of the spleen are subdivided into N_bh_1 and N_bh_ 2 subsets. In comparison with circulating neutrophils N_bh_1 displayed CD15^int^, CD16^int^, CD11b^Hi^, CD24^Hi^, CD27^Hi^, CD40L^Hi^, CD86^Hi^, CD95^Hi^, HLA-I^Hi^, HLA-II^Hi^, CD54^Low^, CD62L ^Low^, CD62P ^Low^ expression whereas N_bh_2 cells had relatively lower expression of CD15 and CD16 [64]. Authors demonstrated that Nbh1 and Nbh2 cells induced higher level of IgM, IgG and IgA in marzinal zone B cells compared to circulating neutrophils [64]. LDNs are documented in both normal physiological state and variety of inflammatory diseases, malignancies and infections. Recently, Blanco-camarillo et al. studied LDNs in healthy individuals displaying normal phenotype as mature neutrophils with CD10^+^ CD11b^+^ CD14^low^ CD15^high^ CD16b^high^ CD62L^+^ CD66b^+^ CXCR4^+^ expression (Fig. 2). These LDNs isolated from healthy volunteers produced increased ROS in response to Phorbol 12-myristate 13-acetate (PMA) and showed higher phagocytic capacity compared to normal neutrophils, however, ability of NETs formation remained unaltered [65].

**Table 1:** Comprehensive overview of neutrophil subsets based on expression of membrane proteins in patho(physiological) states and associated functions

Basis for heterogeneity	Physiological/pathological states	Subsets	Associated function	References
Mouse				
Gene Expression	Granulopoiesis	G0, G1, G2, G3, G4	BM-GMP, proNeu, preNeu, immature Neu and mature Neu	Xie et al. [59]
	Neutrophil aging	CXCR4 (high) CD62L (low)	Increased NETs formation during inflammation	Zhang et al. [31]
	MRSA infection	PMN-1—CD49d^high^CD11b^low^ PMN-2—CD11b^high^CD49d^low^	Immunocompromised hosts may acquire protection against MRSA infection on suppression of PMN-2 or increase in PMN-1 cells	Tsuda et al. [69]
	Multiple sclerosis	ICAM1^+^ extravascular neutrophils	Autoimmune demyelination, MHC class II mediated antigen presentation	Hawkins et al. [118]
Density	Tuberculosis	CD15^high^ CD33^high^ CD66b^high^ CD16^low^	Higher level of ROS and phagocytic capacity	Manna et al. [72]
Humans				
Gene expression	Normal physiological state	CD177	Functionally activated population	Goldschmeding et al. [29]
		OLFM4 neutrophils	Phagocytosis, degranulation, chemotaxis and NETs	Welin et al. [30]
	Granulopoiesis	Lin^−^CD117^+^CD71^+^CD66b^+^	Earliest neutrophil progenitor cells	Dinh et al. [60]
		Nh0, Nh1, Nh2, Nh3	Expression of distinct set of transcription factors	Wigerblad et al. [61]
		Proliferative neutrophil precursor (Preneu)	Differentiated into non-proliferating immature neutrophils and mature neutrophils	Evrard et al. [40]
		GG2, GG3	GG2- granulocytic progenitors GG3- monocytic progenitor cells	Olsson et al., [62]
		G3, G4, G5a, G5b, G5c	Significant alterations in transcriptome between healthy and burn conditions	Huang et al. [63]
	Spatial distribution	N_bh_—CD15^int^, CD16^int^, CD11b^Hi^, CD24^Hi^, CD27^Hi^, CD40L^Hi^, CD86^Hi^, CD95^Hi^, HLA-I^Hi^, HLA-II^Hi^, CD54^Low^, CD62L^Low^, CD62P^Low^	Induced higher level of IgM, IgG and IgA in marzinal zone B cells	Puga et al. [64]
	MRSA infection	CD16^bright^/CD62L^dim^	Ability to contain bacteria intracellularly is decreased	Leliefeld PHC et al. [68]
	Sepsis	CD66b^high^ CD33^low^ CD11b^high^ CD16^±^ CD62L^low^ HLA-DR^low^	Modulates T cell function	Darcy et al. [71]
	*Aspergillus fumigatus* and *Candida albicans* infections	Neutrophilic MDSCs	Impeded T cell and NK cell function	Rieber et al. [73]
	Trauma patients	CD62L^low^CD16^high^	Hyper-segmented nuclei, indicating increased maturation	Hellebrekers et al. [74]
	COVID-19 (pulmonary embolism)	CD16^bright^/CD62L^dim^	Immune-suppressive	Spijkerman et al. [28]
	Gastric cancer patients	CD54^+^ TANs	Supress T cell activity	Wang et al. [102]
	Hepatocellular carcinoma	CD66b + TANs	Increased expression of TNF-α, IL-8, CCL2 and cell-death ligand 1 (PDL1) and decreased CD62L expression	Cheng et al. [103]
	Lung tumors	CD62L^low^CD54^hi^ TANs	Increased production in pro-inflammatory cytokines	Eruslanov et al. [105]
	Colorectal tumor tissues	CD11b^+^CD33^+^CD66b^+^ CD45^+^Lin^−^HLADR^−^	Increased production of ROS and arginase 1	Wu et al. [106]
	Rheumatid arthritis	CD54^high^ and CXCR1^low^	Reverse transmigrated population	Buckley et al. 2006
	Multiple sclerosis	CD43, IL-8R, n-formyl-methionyl-leucyl-phenylalanine and TLR-2	Reduced apoptosis and oxidative burst, increased degranulation and NETs formation	Naegele et al. [119]
Density	Physiological state	CD10^+^ CD11b^+^ CD14^low^ CD15^high^ CD16b^high^ CD62L^+^ CD66b^+^ CXCR4^+^	Increased ROS Higher phagocytic capacity T-cell suppression	Blanco-camarillo et al. [65]
	HIV-1 infected subjects		T-cell suppression	Bowers et al. [17]
	SFTSV		Secreting pro-inflammatory cytokines, damage endothelial cells, phagocytosis of SFTSV	Li et al. [77]
	COVID-19	CD45^+^CD66b^+^CD16^Int^CD44^low^CD11b^Int^	Increased NETs formation	Torrez-Ruiz et al. [90]
	SLE		Production of type I IFNs Prone to produce NETs	Denny et al. [93] Villanueva et al. [12]
	Cancer	CXCR4^high^ CD10 ^low^CD66^high^ PDL-1 ^high/inter^		Shaul et al. [96]

Aged neutrophils in circulation also adds up another layer for heterogeneity and interestingly, aged neutrophils vary in their ability to form NETs. In murine models, aged neutrophils expressing high levels of CXCR4 and decreased levels of CD62L represents an active subset exhibiting increased expression of α_M_β_2_ integrin and NETs formation during inflammation [31]. Microbiota drives neutrophil ageing via Toll-like receptor and myeloid differentiation factor 88-mediated signalling pathways. Depletion of microbiota led to decrease in circulating aged neutrophils and improved the organ damage in endotoxin-induced septic shock mice model [31]. Peng et al. showed that in experimental metastatic cancer models, CXCR4^hi^CD62L^lo^ aged neutrophils displayed increased NETs formation robustly and promoted metastasis [66]. However, proteomics analysis in normal physiological conditions, aged neutrophils showed progressive loss of granular proteins which was associated with decreased ability to form the NETs [67]. Authors exploring endotoxin induced sepsis and acute lung infection (ALI) mouse models demonstrated diurnal differences in NETs forming ability. In ALI models, during night time, neutrophils with abundant granules formed NETs compared to day time which showed progressive loss of granules representing aged counterparts [67]. These studies indicated, ability of aged neutrophils to form NETs may depend on patho(physiological) states. Taken together, above-described studies indicate existence of neutrophil heterogeneity based on differential RNA expression, cell surface markers, density and ageing in normal physiological states and displayed an ability to perform distinct biological functions.

## Neutrophil subtypes and functional relevance during host–pathogen interactions

Immediate recruitment of neutrophils to the infection/inflammatory site is orchestrated by various chemokines such as interferon-gamma (IFN-γ), IL-8, leukotriene and C5a complement [68]. Neutrophils from healthy human individuals upon co-culture with methicillin-resistant Staphylococcus aureus (MRSA) on tissue-like scaffolds showed heterogeneous populations with differential responses in their antimicrobial activity [68]. Neutrophils displaying CD16^bright^/CD62L^dim^ hypersegmented phenotype performed normal phagocytosis but the ability to contain bacteria intracellularly was decreased, on the other hand, CD16^dim^-banded neutrophil subsets effectively restricted MRSA [68]. Tsuda et al. identified two distinct subpopulations of neutrophils in mouse which were referred as PMN-1 and PMN-2 in the context of MRSA infection. PMN-1 exhibited CD49d^high^CD11b^low^ (Fig. 4) phenotype with increased expression of Toll like receptor (TLR) 2, TLR5, TLR4, TLR8, and IL-2 whereas PMN-2 cells displayed CD11b^high^CD49d^low^ with enhanced expression of TLR9, TLR2, TLR7, TLR4 and IL-10. PMN-1 was derived from MRSA resistant hosts, while PMN 2 was obtained from MRSA sensitive hosts [69] Authors suggested that immunocompromised hosts may acquire protection against MRSA infection on suppression of PMN-2 or increase in PMN-1 cells [69]. In the hematopoietic lineage, CMPs differentiates into immune cells along with myeloid-derived suppressor cells (MDSCs). MDSCs are heterogenous population of myeloid origin consisting of myeloid progenitors, immature granulocytes, immature monocytes and immature dendritic cells (Reviewed by Gabrilovich) [70]. Darcy et al. identified neutrophilic MDSCs characterised by the expression of CD66b^high^ CD33^low^ CD11b^high^ CD16^±^ CD62L^low^ HLA-DR^low^ in circulation during sepsis pathogenesis. These MDSCs modulates T cell function and impairs T cell CD3 zeta-chain expression via L-arginine metabolism and contribute to the T cell dysfunction observed in sepsis [71]. In Mycobacterium tuberculosis infection, Manna et al. reported that the frequency of LDNs is associated with severity of tuberculosis. These LDNs displayed CD15^high^ CD33^high^ CD66b^high^ CD16^low^ expression, produced higher level of ROS and phagocytic capacity was increased in comparison with autologous normal density neutrophils [72]. Rieber et al. identified a subset of neutrophilic MDSCs in humans which impeded T cell and NK cell function in *Aspergillus fumigatus* and *Candida albicans* infections. Pathogenic fungi promoted the expression of neutrophilic MDSCs via Dectin-1, a pattern recognition receptor and CARD9, a downstream adaptor protein. Induction of fungal MDSCs is also reliant on pathways downstream of Dectin-1 signalling, such as formation of ROS, caspase-8 activation and IL-1 production [73]. Neutrophils in trauma patients showed distinct subsets characterized by CD62L^low^CD16^high^ levels that displayed hyper-segmented nuclei, indicating increased maturation (Fig. 4) [74]. CD62L^low^CD16^high^ neutrophils have also been observed in bronchoalveolar lavage fluid of infants with various types of viral respiratory infections [75]. CD49d^+^ cysteinyl leukotriene receptor 1 (CysLTR1)^+^ pro-atopic neutrophils which aid in the development of post-viral asthma were increased in nasal lavage in acute respiratory symptoms [76].

Neutrophil subsets referred as LDNs characterized based on buoyancy, have been extensively described in various infections (Fig. 2). Bowers et al. observed a significant increase in LDNs in HIV-1 infected subjects. These LDNs exhibited G-MDSC phenotype along with increased levels of PD-L1 in response to HIV-1 virions and participated in T-cell suppression via PD-L1/PD-1 signalling [17]. Human subjects infected with Severe Fever with Thrombocytopenia Syndrome Virus (SFTSV) displayed LDN subsets characterized by secreting pro-inflammatory cytokines, showed an ability to damage endothelial cells and which were responsible for phagocytosis of SFTSV [77].

Clinical studies in COVID-19 infected subjects showed that the neutrophil-to-lymphocyte ratio (NLR) correlated with disease severity and NLR was characterized by a reduced lymphocyte count and increased neutrophils [78–81]. Pre-clinical and clinical models have demonstrated two independent mechanisms for neutrophil activation during COVID-19 infections. SARS-CoV-2 infected alveolar epithelial cells release abundant levels of IL-6, IL-8, CXCL1 and CXCL2 resulting in the recruitment of neutrophils and in-turn, these activated neutrophils form extracellular traps and contribute to organ damage [82, 83]. On the other hand, Veras et al. showed higher concentrations of NETs components in plasma, tracheal aspirates and lung autopsies in COVID-19 subjects. Mechanistically, authors demonstrated SARS-CoV-2 induced NETosis in healthy neutrophils in PAD-4 dependent manner [84]. Masso-silva et al. showed significantly higher levels of NETs in plasma and tracheal aspirate of patients hospitalized with COVID-19 and spontaneous NETs production was observed in SARS-CoV-2-infected lung airways and alveoli [85, 86]. Neutrophil heterogeneity based on different densities, maturity and expression levels of surface markers have been observed in COVID-19 pathogenesis [28, 87]. Carissimo et al. demonstrated a dramatic increase in immature neutrophils in COVID-19 patients which strongly correlated with the level of increased IL-6 and Hepatocyte growth factor (IP-10) which are crucial in driving cytokine storm and COVID-19 severity [88, 89]. A strong correlation was observed between an increase in LDNs and disease severity in COVID-19 patients along with ability to produce NETs in comparison to healthy individuals [87, 90]. LDNs expressing CD45^+^CD66b^+^CD16^Int^CD44^low^CD11b^Int^ were observed in severe COVID-19 patients with increased cytokine production, spontaneous NETs formation and enhanced phagocytic capacity. Increased immuno-suppressive CD16^bright^/CD62L^dim^ neutrophils in patients with pulmonary embolism on the day of ICU admission were also identified [28]. Schulte-Schrepping et al. observed increased expression of programmed death ligand (PD-L) 1 on immature neutrophils which suppressed T cells in COVID-19 patients [91].

## Neutrophil Subsets in pathologies associated with sterile inflammation

### Systemic lupus erythematosus (SLE)

LDNs have also been attributed in SLE pathogenesis (Fig. 2) [92]. LDNs display pro-inflammatory phenotypes with increased production of IFN-g, TNF-α, and type I IFNs in SL and cause considerable endothelial cell cytotoxicity [94]. Increased production of type I IFNs by LDNs prevented the differentiation of endothelial progenitor cells to form mature endothelial cells [93]. In SLE subjects, LDNs were constitutively active, express elevated alarmins and immuno-stimulatory bactericidal proteins and were more prone to produce NETs leading to endothelial cell toxicity [12]. SLE patients showed elevated levels of LDNs, positively correlating with disease progression [94]. Noncalcified plaque burden (NCB) has been associated with LDNs in SLE patients and these activated LDNs contribute instability of coronary plaques [26].

### Paradoxical effects of neutrophil sub-types in the pathogenesis of cancer

**Fig. 3:**
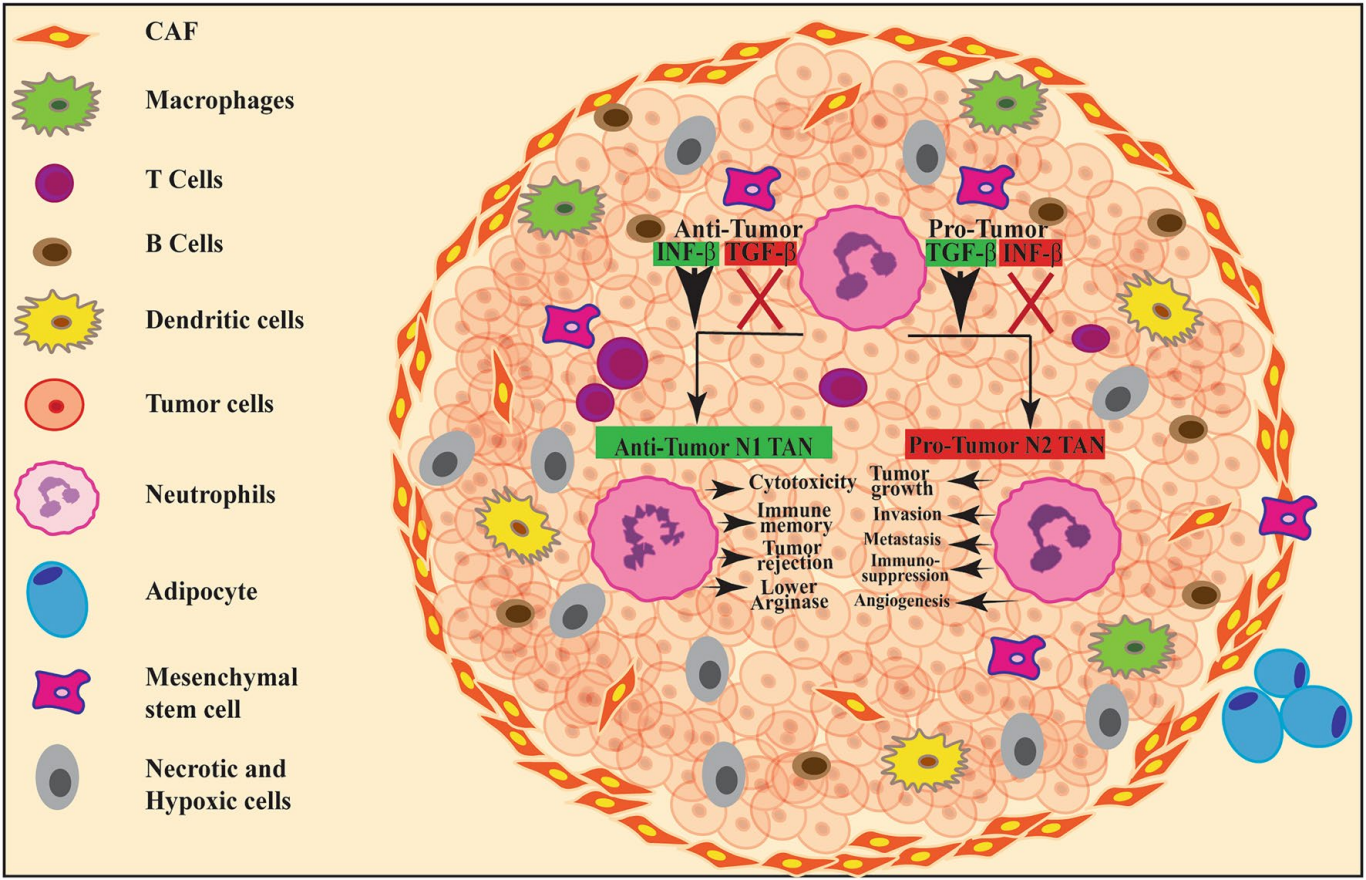
Paradoxical role of neutrophils in tumors. In tumor microenvironment, Anti-tumor N1 TANs display cytotoxicity, immune memory, rejection of tumour, producing lower arginase. Whereas pro-tumor N2 TANs gets involve in invasion, metastasis, immune suppression and angiogenesis leading to the progression of tumor

Neutrophils play an important and divisive role in the progression of solid tumors and the spread of malignancies. Neutrophil subpopulations have been identified with contrasting activities during tumor inflammation. Peripheral blood of cancer patients and experimental animals with tumors exhibit three subsets of neutrophils based on their density which includes high-density neutrophils (HDNs), LDNs and granulocytic-MDSCs. LDNs showed mature morphology and segmented nucleus whereas G-MDSCs display immature morphology with the expression of CD33^+^/CD14^−^/CD66b^+^/CD15^+^/CD11b^+^/ HLA-DR^−^/ cell surface phenotype [95]. In subjects with advanced lung tumors, Shaul et al. using mass cytometry showed that 50% of LDNs expressed CXCR4^high^ CD10 ^low^CD66^high^ PDL-1 ^high/inter^ indicating heterogeneity within LDN population [96]. Tumor associated neutrophils (TAN) showed hypersegmented nuclei with an increased ability to kill tumor cells and were characterized by elevated production of pro-inflammatory cytokines as shown in Fig. 3. TANs are differentiated into anti-tumor neutrophils (N1) and pro-tumor neutrophils (N2). N1 neutrophils exhibit anti-tumor properties. N2 neutrophils differentiated in TGF-β dependent manner show immature phenotype, increased arginase activity, inhibit T cell proliferation and promote tumor growth (Fig. 3) [97]. Zou et al. observed increased neutrophil counts in circulation, infiltration of TANs into a tumor and enhanced transition of TANs into N2 phenotype in vitro and in vivo models. IL-35, which is highly expressed in tumor tissue is responsible for the polarization of N2 phenotype and increased the neutrophil numbers in a tumor. IL-35 dependent IL-17 production served as a pro-tumorigenic factor which in turn elevated the expression of G-CSF and IL-6 leading to increased recruitment of neutrophils into the tumour microenvironment. IL-35 depleted the expression of TNF-related apoptosis-inducing ligand (TRAIL) to increase the pro-angiogenic properties of neutrophils, and enhanced tumor growth and progression [98]. NK cells modulate the inflammatory property of neutrophils via IFN-γ-stimulated pathway to inhibit the expression of vascular endothelial growth factor-A (VEGF-A) which promoted angiogenesis and subsequent tumor growth in a rodent model [99]. Massena et al. identified pro-angiogenic subtype of neutrophils expressing VEGFR1^+^, CxCR4^+^, and CD49d^+^. Authors demonstrated that CD49d played a crucial role in the recruitment of neutrophils in response to VEGF-A and targeting this surface marker reduced the recruitment of proangiogenic neutrophils to hypoxic tissue [100]. Transcriptome analysis of three neutrophil subpopulations including naïve neutrophils, TANs and G-MDSCs revealed that TANs represented a distinct RNA profile [101]. TANs showed reduced expression of genes associated with oxidative burst while pro-inflammatory genes and antigen presenting complex genes were elevated [98]. In Gastric cancer patients, CD54^+^ TANs expressing high level of PD-L1, supress T cell activity via PD-1-PD-L-1 pathway and correlates with poor prognosis [102]. In hepatocellular carcinoma (HCC), CD66b + TANs exhibited increased expression of TNF-α, IL-8, CCL2 and cell-death ligand 1 (PDL1) and decreased CD62L expression. Prolonged survival and functional activity of TANs might be due to the higher expression of PDL1 through IL-6-STAT3-PDL1 signalling mechanism [103]. In melanoma models Huh et al. demonstrated IL-8 expressed by tumor cells increased the expression of β2 integrin and subsequently enhanced the interaction between neutrophils and melanoma cells. This interaction allowed the melanoma cells to transmigrate through the endothelium. IL-8 also helped in the retention of neutrophils in lung tissue [104]. These findings suggested that TANs are polarized from N1 phenotype to N2 phenotype depending on the tumor microenvironment indicting the plasticity and heterogeneity of neutrophils. TANs isolated from human lung tumors exhibited CD62L^low^CD54^hi^ phenotype with increased production in pro-inflammatory cytokines which enhanced T cell function and production of IFN-γ [105]. In colorectal tumor tissues, TANs exhibited similar morphology as normal neutrophils and displayed CD11b^+^CD33^+^CD66b^+^ CD45^+^Lin^−^HLADR^−^ phenotype with increased production of ROS and arginase 1 [106]. MDSCs observed in tumors of lung, bladder, head and neck exhibited low density phenotype, altered expression of cell surface markers, impaired effector functions and prolonged survival in comparison with normal neutrophils. These neutrophilic MDSCs lacking in chemokine receptors CXCR1 and CXCR2 which are essential for the extravasation of neutrophils led to reduced chemotaxis towards the tumour environment [107].

### Neutrophil subtypes in autoimmune disorders

Mounting evidence implies that neutrophils play a crucial role in the course and severity of numerous autoimmune disorders [108]. Neutrophils in autoimmune disorders exhibit pro-inflammatory properties characterized by increased production of inflammatory mediators which subsequently lead to the production of autoantibodies and prime other leukocytes [109]. In SLE, apoptotic neutrophils are increased in correlation with pathological activity and anti-double-stranded DNA (anti-dsDNA) antibody levels [110]. Abnormal neutrophil adhesion and chemotaxis has been observed in rheumatoid arthritis [111]. Neutrophil heterogeneity and their functional relevance in various autoimmune disorders are discussed below.

### Rheumatoid arthritis

Rheumatoid arthritis (RA) is a chronic autoimmune illness that causes inflammation in the joints and cartilage tissues. Peripheral blood neutrophils of RA subjects show distinct functionality from those in healthy individuals and are constitutively activated to produce ROS. Transcriptome analysis of RA neutrophils showed elevated expression of myeloblastin, TNF-α and membrane-bound receptor activator of nuclear factor κB (NF-κB) ligand (RANKL) [112]. Primed neutrophils from RA secreted cytokines such as B cell-activating factor (BAFF) and RANKL which activated B cells, osteoclasts and CD4^+^ T cells [113, 114]. Cytokines including GM-CSF, IL-8 and TNF-α delayed neutrophil apoptosis and primed neutrophils to release granules in the synovial cavity [115]. RA neutrophils expressed high concentrations of elastase, gelatinase and collagenase responsible for tissue and cartilage damage [116]. Buckley et al., identified a phenotypically and functionally distinct subpopulation of neutrophils with the expression of CD54^high^ and CXCR1^low^ in RA individuals which showed a reverse transmigrated population [115]. These cells were different from tissue resident neutrophils which expressed CD54^low^ and CXCR1^low^ [115].

### Multiple sclerosis

**Fig. 4:**
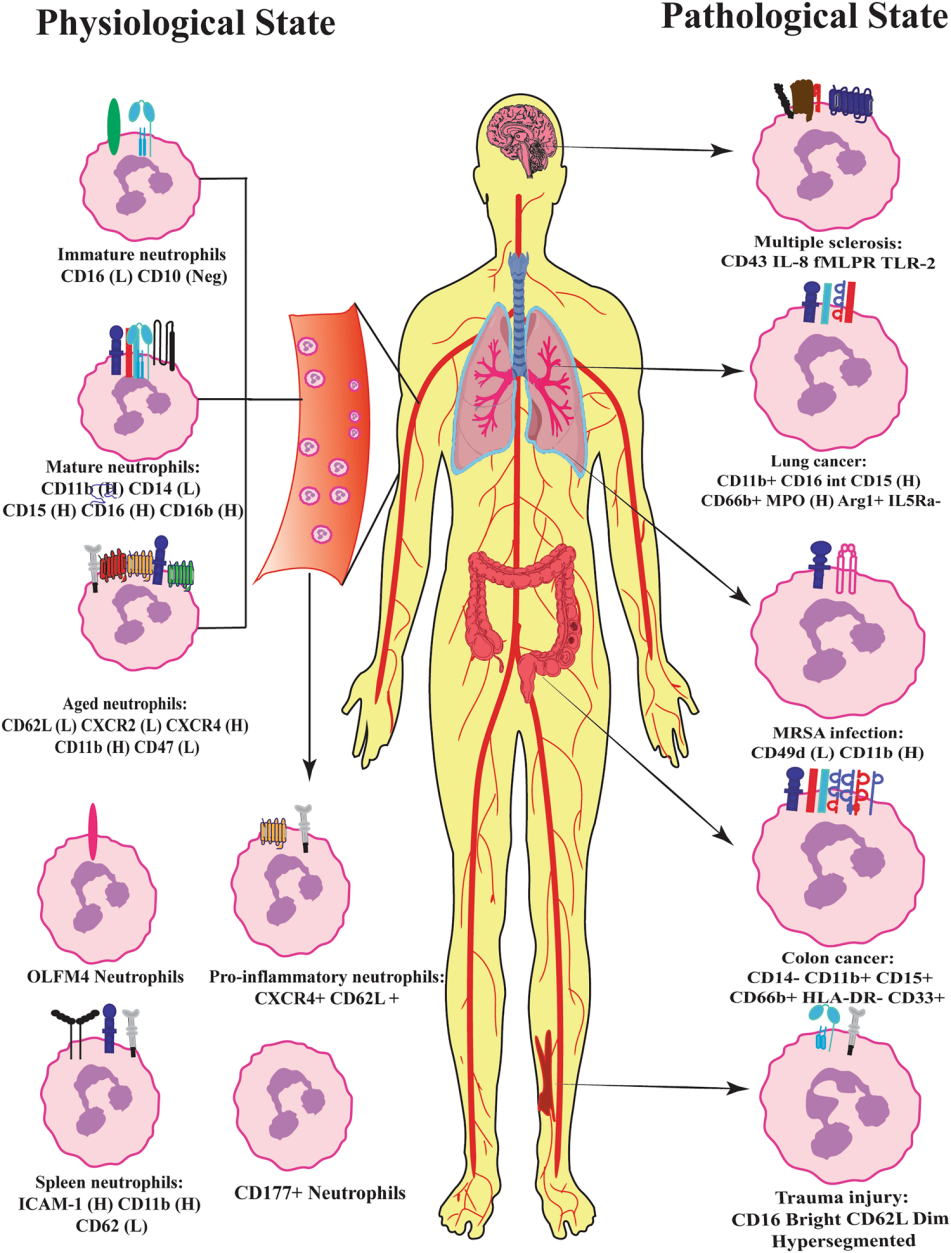
Neutrophil subsets in physiological and pathological states. The illustration shows various neutrophil subtypes characterized by differential expression of surface markers identified in steady state and diseases

With an estimated prevalence of 2.5 million affected subjects globally, multiple sclerosis (MS) is the most common immune-mediated inflammatory illness affecting the central nervous system (CNS) [117]. Extravascular neutrophils expressing ICAM1^+^ in animal models of autoimmune encephalomyelitis (EAE) for multiple sclerosis were identified by Hawkins et al. (Fig. 4) [117]. These neutrophil subsets acquired macrophage like properties by showing MHC class II mediated antigen presentation and expressed aspartic peptidase retroviral-like 1 (ASPRV1, also known as SASPase) enzyme involved in autoimmune demyelination [118]. Neutrophils isolated from MS subjects expressed higher levels of CD43, IL-8R, n-formyl-methionyl-leucyl-phenylalanine and TLR-2 forming a distinct phenotype as shown in Fig. 4. These cells exhibited altered functionality with reduced apoptosis and oxidative burst, increased degranulation and NETs formation [119].

### Systemic inflammatory response syndrome (SIRS)

Systemic inflammatory response syndrome (SIRS) is commonly observed in patients with severe burn injuries, pancreatitis, major surgery and polytrauma [69]. In addition to normal neutrophils (PMN), two other subsets PMN-I and PMN-II have been identified in SIRS patients. These cells differ in the expression of chemokine and cytokines. PMN-I displayed the expression of IL-12/CCL3 whereas PMN-II expressed IL-10/CCL2. PMN-I and PMN-II showed differential expression of toll-like receptors where PMN-I were characterized by expression of TLR8/TLR5/TLR4/TLR2, whereas PMN-II expressed TLR9/TLR7/TLR4/TLR2. These cells also differed in the distribution of cell surface markers, PMN-I displayed CD11b^−^CD49d^+^ and PMN-II exhibited CD11b^+^CD49d^−^ [69].^.^

### Current therapeutic approaches targeting neutrophils

Activated neutrophils are involved in many acute and chronic inflammatory diseases such as autoimmune disorders, cardiovascular diseases (thrombosis and atherosclerosis), respiratory diseases (COPD, asthma and ARDS), [120], neurological disorder (Alzheimer’s and multiple sclerosis) [121, 122], skin diseases (Behçet’s disease and psoriasis) [123, 124], metabolic diseases (obesity and diabetes mellitus) [125, 126] and gastrointestinal diseases (inflammatory bowel and autoimmune hepatitis) [127]. Although, neutrophils possess a beneficial role in eliminating infections, over-functioning or failure to post-infection clearance of these cells causes significant tissue damage in aforementioned diseases. Hence, over the years, several studies have attempted to modulate over-functioning of neutrophils through pharmacological strategies. Wu et al. observed that CXCR2 antagonist SB225002 and theophylline induce a significant decrease in neutrophil viability and accelerated neutrophil apoptosis [128]. Food and Drug Administration (FDA) approved drug N-Acetylcysteine (Mucolytic drug) reduced ROS production in vitro and triggered a self-sustaining phlogogenic loop in the respiratory system [129]. Randomized, placebo-controlled, human study showed that AZD7986 (DPP1 inhibitor) inhibited whole blood neutrophil elastase activity [130]. Ali et al. reported that selective agonism of the adenosine A_2A_ receptor (CGS21680) suppresses antiphospholipid antibodies -mediated NETosis in protein kinase A-dependent fashion. CGS21680 also reduced thrombosis in the inferior venae cavae in in vivo models [131]. Lotamilast is a moderately potent PDE4 inhibitor (IC_50_ = 2.8 nM) that effectively suppressed LPS induced neutrophilic pulmonary inflammation in mouse models [132]. Aikawa et al. demonstrated a clinical trial to treat ARDS by using inhibitor of neutrophil elastase Sivelestat (Elaspol, ONO 5046) [133].

NETs forming ability by neutrophils significantly varies during life time. Lipp et al. demonstrated that NETs forming ability was found reduced in infants (35–39 weeks) in comparison with healthy adults [134]. Neonates showed inability to form NETs because of expression of Neonatal NET-inhibitory factor (nNIF) that inhibited NETs formation by targeting the activity of peptidyl arginine deiminase 4 (PAD4), histone citrullination and nuclear condensation [135]. Further, nNIF administration was explored for inhibiting NETs in pathological states. nNIF improved the mortality in the CLP model of polymicrobial sepsis [135]. In murine model of ischemic stroke, nNIF delivery decreased ischemic stroke brain injury and related mortality [136]. Prostaglandins (PGE2) synthesized endogenously in arachidonic acid pathway inhibits PMA induced NETs formation through the activation of protein kinase A and cyclic AMP [137]. Tilgner et al. showcased the property of aspirin to reduce the production of NETs in C57BL/6 mice induced with acute lung injury (ALI) [138]. Li et al. showed the delay in the progression of multiple myeloma by inhibiting NETs formation upon targeting PAD4 using BMS-P5 [139]. In cystic fibrosis (CF), administration of rhDNase and airway clearance therapy are most frequently used techniques to mobilize sputum which contains large amount of extracellular DNA released by leukocyte specifically neutrophils. The rhDNase cleaves extracellular DNA, reduces viscoelasticity of sputum and mobilize sputum [140]. Authors involving 43 subjects with CF, demonstrated rhDNases improved the sputum mobilization [140]. A study involving 968 subjects with CF, demonstrated administration of rhDNases showed reduced respiratory exacerbations [141]. It is known that Gasdermin-D (GSDMD) is a key mediator of NETosis and a study consisting of 63 hospitalized patients with moderate and severe COVID-19 revealed higher expression of GSDMD genes [142]. In a mouse model of SARS-CoV-2 infection, the treatment with disulfiram (GSDMD inhibitor) inhibited NETs release and reduced organ damage [142].

In recent years, selective targeting of activated neutrophils in diseased models have been demonstrated. Employing nanotechnology Wang et al. delivered piceatannol, a small molecule that blocks β2 integrin pathway to prevent vascular inflammation through albumin nanoparticles which selectively targeted highly activated neutrophils attached to endothelium preventing neutrophil infiltration in murine models [143]. Bachmaier et al. identified two subsets of neutrophils based on endocytosis of albumin nanoparticles (ANP) (ANP^high^ and ANP^low^). ANP^high^ neutrophils produced an inordinate amount of ROS and inflammatory cytokines. Authors targeted these ANP^high^ neutrophils with ANPs loaded with piceatannol, a spleen tyrosine kinase (Syk) inhibitor to reduce the inflammation in sepsis and preserved neutrophilic host defense function in cecal ligation and puncture (CLP) mice model [144]. Activated neutrophils were also targeted utilizing α1-antitrypsin-derived peptide (surface decoration) to confer binding specificity to neutrophil elastase which enabled specific anchorage of nanoparticles to activated neutrophils [145]. In murine models of deep vein thrombosis, delivery of hydrochloroquine encapsulated in nanoparticles resulted in significantly smaller thrombi compared to either control or hydrocholoriquine alone [145].

## Concluding remarks and future prospects

Genetic and epigenetic analysis revealed subsets of neutrophils expressing distinct transcriptional networks and concomitant differential gene expression and further displayed heterogeneous functions [24, 61, 62]. Existing data on neutrophil subtypes are based on transcriptome analysis, membrane markers and density. Neutrophils are terminally differentiated cells with decreased ability for transcription and also translation of nascent proteins. Studies have demonstrated transcriptional firing in response to stimuli such as LPS, PMA, A23187 and ionomycin [146]. However, proportions of these mRNA translated to proteins was unclear [146]. Recent studies exploring RNA sequencing has demonstrated existence of neutrophil subsets in both physiological and pathological states which may also indicate formation of neutrophil subsets in bone marrow itself and released to peripheral tissue with a pre-determined function [40, 59, 60, 62]. Further, these subsets may acquire different states by post-translational modifications and metabolic states in response to patho(physiological) stimuli. Several layers of heterogeneity have been attributed to the formation of neutrophil subpopulations influenced by development, environment and activation state in both steady state and pathological conditions. Upstream pathogenic inducers and signalling mediators significantly vary among diseases and hence contribute to temporal diversity and dynamics of pathologically specific subsets and further attribute to the severity and duration of diseases. Hence, gene expression/protein/post-translational modifications and metabolic intermediates also display disease specific profiles in activated neutrophils. Accordingly, insights into cellular and molecular mechanisms regulating functions of activated neutrophil subtypes/sub population may facilitate designing therapeutic targets specific to diseases. Moreover, inactive neutrophils are also gaining attention in several pathological conditions. Hence, designing anti-neutrophil therapies requires careful tailoring by only targeting subset of activated neutrophils and simultaneously maintaining their normal functioning. Hence, ‘pan-anti-neutrophil’ therapies and pharmacological blocking/activating specific signalling pathways might not be beneficial as pathological stimuli and associated signalling pathways may vary in neutrophil subsets during their activation in different diseases [33]. Inhibition of over functioning of neutrophils and simultaneously maintaining neutrophil homeostasis and restoring organ function may serve as a potential therapeutic strategy. For example, our earlier studies in the context of T2D show high glucose induces constitutive NETosis and leads to reduced response to infection [4]. Hence inhibition of glucose induced NETs formation and restoring the anti-microbial function of neutrophils to fight against infection may help in the clinical management of T2D associated infections. Generally, it is also experienced that neutrophils are activated while isolation which may be due to magnetic beads, density gradient compounds and mere centrifugation. Hence, full-fledged clinical studies using systems biology approaches involving the identification of epigenetic/genetic signatures along with transcriptomic, proteomic and metabolic profiles in neutrophil subsets in disease specific manner may help in a better understanding of neutrophil biology.

## Data Availability

Not applicable; all information is gathered from published articles.

## References

[1] Kolaczkowska E, Kubes P (2013). Neutrophil recruitment and function in health and inflammation. Nat Rev Immunol.

[2] Aroca-Crevillén A, Adrover JM, Hidalgo A (2020). Circadian features of neutrophil biology. Front Immunol.

[3] Suratt BT, Young SK, Lieber J, Nick JA, Henson PM, Scott Worthen G (2001). Neutrophil maturation and activation determine anatomic site of clearance from circulation. Am J Physiol Lung Cell Mol Physiol.

[4] Joshi MB, Ahamed R, Hegde M, Nair AS, Ramachandra L, Satyamoorthy K (2020). Glucose induces metabolic reprogramming in neutrophils during type 2 diabetes to form constitutive extracellular traps and decreased responsiveness to lipopolysaccharides. Biochim Biophys Acta Mol Basis Dis.

[5] Adrover JM, del Fresno C, Crainiciuc G (2019). A neutrophil timer coordinates immune defense and vascular protection. Immunity.

[6] Brinkmann V, Reichard U, Goosmann C (2004). Neutrophil extracellular traps kill bacteria. Science.

[7] Díaz-Godínez C, Carrero JC (2019). The state of art of neutrophil extracellular traps in protozoan and helminthic infections. Biosci Rep.

[8] Su Y, Gao J, Kaur P, Wang Z (2020). Neutrophils and macrophages as targets for development of nanotherapeutics in inflammatory diseases. Pharmaceutics.

[9] Fadok VA, Bratton DL, Konowal A, et al. macrophages that have ingested apoptotic cells in vitro inhibit proinflammatory cytokine production through autocrine/paracrine mechanisms involving TGF−, PGE2, and PAF. J Clin Invest. 1998;15;101(4):890–8. 10.1172/JCI1112 10.1172/JCI1112 PMC508637 9466984

[10] Garcia-Romo GS, Caielli S, Vega B (2011). Netting neutrophils are major inducers of type I IFN production in pediatric systemic lupus erythematosus. Sci Transl Med..

[11] Lande R, Ganguly D, Facchinetti V (2011). Neutrophils activate plasmacytoid dendritic cells by releasing self-DNA-peptide complexes in systemic lupus erythematosus. Sci Transl Med..

[12] Villanueva E, Yalavarthi S, Berthier CC (2011). Netting neutrophils induce endothelial damage, infiltrate tissues, and expose immunostimulatory molecules in systemic lupus erythematosus. J Immunol.

[13] Massena S, Christoffersson G, Vågesjö E (2015). Identification and characterization of VEGF-A-responsive neutrophils expressing CD49d, VEGFR1, and CXCR4 in mice and humans. Blood.

[14] Wingender G, Hiss M, Engel I (2012). Neutrophilic granulocytes modulate invariant NKT cell function in mice and humans. J Immunol.

[15] Barrientos L, Bignon A, Gueguen C (2014). Neutrophil extracellular traps downregulate lipopolysaccharide-induced activation of monocyte-derived dendritic cells. J Immunol.

[16] Scapini P, Nardelli B, Nadali G (2003). G-CSF-stimulated neutrophils are a prominent source of functional BLyS. J Exp Med.

[17] Bowers NL, Helton ES, Huijbregts RPH (2014). Immune suppression by Nneutrophils in HIV-1 infection: role of PD-L1/PD-1 pathway. PLoS Pathog.

[18] Martinod K, Demers M, Fuchs TA (2013). Neutrophil histone modification by peptidylarginine deiminase 4 is critical for deep vein thrombosis in mice. Proc Natl Acad Sci U S A.

[19] Guglietta S, Chiavelli A, Zagato E (2016). Coagulation induced by C3aR-dependent NETosis drives protumorigenic neutrophils during small intestinal tumorigenesis. Nat Commun.

[20] Boone BA, Orlichenko L, Schapiro NE (2015). The receptor for advanced glycation end products (RAGE) enhances autophagy and neutrophil extracellular traps in pancreatic cancer. Cancer Gene Ther.

[21] Joshi MB, Baipadithaya G, Balakrishnan A (2016). Elevated homocysteine levels in type 2 diabetes induce constitutive neutrophil extracellular traps. Sci Rep.

[22] Joshi MB, Lad A, Bharath Prasad AS (2013). High glucose modulates IL-6 mediated immune homeostasis through impeding neutrophil extracellular trap formation. FEBS Lett.

[23] Khandpur R, Carmona-Rivera C, Vivekanandan-Giri A (2013). NETs are a source of citrullinated autoantigens and stimulate inflammatory responses in rheumatoid arthritis. Sci Transl Med..

[24] Khoyratty TE, Ai Z, Ballesteros I (2021). Distinct transcription factor networks control neutrophil-driven inflammation. Nat Immunol.

[25] Ballesteros I, Rubio-Ponce A, Genua M (2020). Co-option of neutrophil fates by tissue environments. Cell.

[26] Carlucci PM, Purmalek MM, Dey AK (2018). Neutrophil subsets and their gene signature associate with vascular inflammation and coronary atherosclerosis in lupus. JCI Insight.

[27] Sagiv JY, Michaeli J, Assi S (2015). Phenotypic diversity and plasticity in circulating neutrophil subpopulations in cancer. Cell Rep.

[28] Spijkerman R, Jorritsma NKN, Bongers SH (2021). An increase in CD62Ldim neutrophils precedes the development of pulmonary embolisms in COVID-19 patients. Scand J Immunol.

[29] Goldschmeding R, van Dalen CM, Faber N (1992). Further characterization of the NB 1 antigen as a variably expressed 56–62 kD GPI-linked glycoprotein of plasma membranes and specific granules of neutrophils. Br J Haematol.

[30] Welin A, Amirbeagi F, Christenson K (2013). The human neutrophil subsets defined by the presence or absence of OLFM4 both transmigrate into tissue in vivo and give rise to distinct NETs in vitro. PLoS ONE.

[31] Zhang D, Chen G, Manwani D (2015). Neutrophil ageing is regulated by the microbiome. Nature.

[32] Douda DN, Khan MA, Grasemann H, Palaniyar N (2015). SK3 channel and mitochondrial ROS mediate NADPH oxidase-independent NETosis induced by calcium influx. Proc Natl Acad Sci U S A.

[33] Thimmappa PY, Nair AS, Najar MA (2022). Quantitative phosphoproteomics reveals diverse stimuli activate distinct signaling pathways during neutrophil activation. Cell Tissue Res.

[34] Baum CM, Weissman IL, Tsukamoto AS (1992). Isolation of a candidate human hematopoietic stem-cell population. Proc Natl Acad Sci U S A.

[35] Cvejic A (2016). Mechanisms of fate decision and lineage commitment during haematopoiesis. Immunol Cell Biol.

[36] Nandakumar SK, Ulirsch JC, Sankaran VG (2016). Advances in understanding erythropoiesis: Evolving perspectives. Br J Haematol.

[37] von Vietinghoff S, Ley K (2008). Homeostatic regulation of blood neutrophil counts. J Immunol.

[38] Kim MH, Yang D, Kim M (2017). A late-lineage murine neutrophil precursor population exhibits dynamic changes during demand-adapted granulopoiesis. Sci Rep.

[39] Terstappen LW, Safford M, Loken MR (1990). Flow cytometric analysis of human bone marrow. III Neutrophil maturation Leukemia.

[40] Evrard M, Kwok IWH, Chong SZ, Teng KWW, Becht E, Chen J (2018). Developmental analysis of bone marrow neutrophils reveals populations specialized in expansion, trafficking, and effector functions. Immunity.

[41] Anderson KL, Smith KA, Pio F (1998). Neutrophils deficient in PU.1 do not terminally differentiate or become functionally competent. Blood.

[42] Zhang D-E, Zhang PU, Wang N-D (1997). Absence of granulocyte colony-stimulating factor signaling and neutrophil development in CCAAT enhancer binding protein-deficient mice. Proc Natl Acad Sci U S A.

[43] Growney JD, Shigematsu H, Li Z (2005). Loss of Runx1 perturbs adult hematopoiesis and is associated with a myeloproliferative phenotype. Blood.

[44] Eash KJ, Greenbaum AM, Gopalan PK, Link DC (2010). CXCR2 and CXCR4 antagonistically regulate neutrophil trafficking from murine bone marrow. J Clin Investig.

[45] Furze RC, Rankin SM (2008). The role of the bone marrow in neutrophil clearance under homeostatic conditions in the mouse. FASEB J.

[46] Semerad CL, Liu F, Gregory AD, Stumpf K, Link DC (2002). G-CSF is an essential regulator of neutrophil trafficking from the bone marrow to the blood. Immunity.

[47] Semerad CL, Christopher MJ, Liu F, Short B, Simmons PJ, Winkler I (2005). G-CSF potently inhibits osteoblast activity and CXCL12 mRNA expression in the bone marrow. Blood.

[48] Méndez-Ferrer S, Lucas D, Battista M, Frenette PS (2008). Haematopoietic stem cell release is regulated by circadian oscillations. Nature.

[49] De Filippo K, Rankin SM (2018). CXCR4, the master regulator of neutrophil trafficking in homeostasis and disease. Eur J Clin Invest.

[50] Casanova-Acebes M, Nicolás-Ávila JA, Yao Li JL (2018). Neutrophils instruct homeostatic and pathological states in naive tissues. J Exp Med.

[51] Coralie Burdon PC, Bridger G, Gutierrez-Ramos JC, Williams TJ, Rankin SM (2003). Chemokines acting via CXCR2 and CXCR4 control the release of neutrophils from the bone marrow and their return following senescence. Immunity.

[52] Shi J, Gilbert GE, Kokubo Y, Ohashi T (2001). Role of the liver in regulating numbers of circulating neutrophils. Blood.

[53] Stark MA, Huo Y, Burcin TL (2005). Phagocytosis of apoptotic neutrophils regulates granulopoiesis via IL-23 and IL-17. Immunity.

[54] Hirai H, Zhang P, Dayaram T (2006). C/EBPβ is required for “emergency” granulopoiesis. Nat Immunol.

[55] Hirai H, Yokota A, Tamura A (2015). Non-steady-state hematopoiesis regulated by the C/EBPβ transcription factor. Cancer Sci.

[56] Walker F, Zhang H-H, Matthews V (2008). IL6/sIL6R complex contributes to emergency granulopoietic responses in G-CSF– and GM-CSF–deficient mice. Blood.

[57] Kwak HJ, Liu P, Bajrami B (2015). Myeloid cell-derived reactive oxygen species externally regulate the proliferation of myeloid progenitors in emergency granulopoiesis. Immunity.

[58] Lawrence SM, Corriden R, Nizet V (2018). The ontogeny of a neutrophil: mechanisms of granulopoiesis and homeostasis. Microbiol Mol Biol Rev.

[59] Xie X, Shi Q, Wu P (2020). Single-cell transcriptome profiling reveals neutrophil heterogeneity in homeostasis and infection. Nat Immunol.

[60] Dinh HQ, Eggert T, Meyer MA (2020). Coexpression of CD71 and CD117 identifies an early unipotent neutrophil progenitor population in human bone marrow. Immunity.

[61] Wigerblad G, Cao Q, Brooks S (2022). Single-cell analysis reveals the range of transcriptional states of circulating human neutrophils. J Immunol.

[62] Olsson A, Venkatasubramanian M, Chaudhri VK (2016). Single-cell analysis of mixed-lineage states leading to a binary cell fate choice. Nature.

[63] Huang J, Zhu Z, Ji D (2022). Single-cell transcriptome profiling reveals neutrophil heterogeneity and functional multiplicity in the early stage of severe burn patients. Front Immunol.

[64] Puga I, Cols M, Barra CM (2012). B cell-helper neutrophils stimulate the diversification and production of immunoglobulin in the marginal zone of the spleen. Nat Immunol.

[65] Blanco-Camarillo C, Alemán OR, Rosales C (2021). Low-density neutrophils in healthy individuals display a mature primed phenotype. Front Immunol.

[66] Peng Z, Liu C, Victor AR, Cao DY, Veiras LC, Bernstein EA (2021). Tumors exploit CXCR4hiCD62Llo aged neutrophils to facilitate metastatic spread. Oncoimmunology..

[67] Adrover JM, Aroca-Crevillén A, Crainiciuc G, Ostos F, Rojas-Vega Y, Rubio-Ponce A (2020). Programmed ‘disarming’ of the neutrophil proteome reduces the magnitude of inflammation. Nat Immunol.

[68] Leliefeld PHC, Pillay J, Vrisekoop N (2018). Differential antibacterial control by neutrophil subsets. Blood Adv.

[69] Tsuda Y, Takahashi H, Kobayashi M (2004). Three different neutrophil subsets exhibited in mice with different susceptibilities to infection by methicillin-resistant *Staphylococcus aureus*. Immunity.

[70] Gabrilovich DI, Nagaraj S (2009). Myeloid-derived suppressor cells as regulators of the immune system. Nat Rev Immunol.

[71] Darcy CJ, Minigo G, Piera KA (2014). Neutrophils with myeloid derived suppressor function deplete arginine and constrain T cell function in septic shock patients. Crit Care.

[72] La Manna MP, Orlando V, Paraboschi EM, Tamburini B, Di Carlo P, Cascio A (2019). *Mycobacterium tuberculosis* drives expansion of low-density neutrophils equipped with regulatory activities. Front Immunol.

[73] Rieber N, Singh A, Öz H (2015). Pathogenic fungi regulate immunity by inducing neutrophilic myeloid-derived suppressor cells. Cell Host Microbe.

[74] Hellebrekers P, Hesselink L, Huisman A (2020). Recognizing the mobilization of neutrophils with banded nuclei early after trauma. Int J Lab Hematol.

[75] Cortjens B, Ingelse SA, Calis JC (2017). Neutrophil subset responses in infants with severe viral respiratory infection. Clin Immunol.

[76] Cheung DS, Sigua JA (2018). Cysteinyl leukotriene receptor 1 expression identifies a subset of neutrophils during the antiviral response that contributes to postviral atopic airway disease. J Allergy Clin Immunol.

[77] Li Y, Li H, Wang H (2019). The proportion, origin and pro-inflammation roles of low density neutrophils in SFTS disease. BMC Infect Dis.

[78] Toori KU, Qureshi MA, Chaudhry A, Safdar MF (2021). Neutrophil to lymphocyte ratio (Nlr) in covid-19: A cheap prognostic marker in a resource constraint setting. Pak J Med Sci..

[79] Ciccullo A, Borghetti A, Zileri Dal Verme L, et al. Neutrophil-to-lymphocyte ratio and clinical outcome in COVID-19: a report from the Italian front line. Int J Antimicrob Agents. 2020. 10.1016/j.ijantimicag.2020.106017. 10.1016/j.ijantimicag.2020.106017 PMC7211594 32437920

[80] Gelzo M, Cacciapuoti S, Pinchera B (2021). Prognostic role of neutrophil to lymphocyte ratio in COVID-19 patients: still valid in patients that had started therapy?. Front Public Health.

[81] Nair PR, Maitra S, Ray BR (2020). Neutrophil-to-lymphocyte ratio and platelet-to-lymphocyte ratio as predictors of the early requirement of mechanical ventilation in covid-19 patients. Indian J Crit Care Med.

[82] Gajewski T, Rouhani S, Trujillo J (2021). Severe COVID-19 infection is associated with aberrant cytokine production by infected lung epithelial cells rather than by systemic immune dysfunction. Res Sq..

[83] Leppkes M, Knopf J, Naschberger E (2020). Vascular occlusion by neutrophil extracellular traps in COVID-19. EBioMedicine.

[84] Veras FP, Pontelli MC, Silva CM (2020). SARS-CoV-2-triggered neutrophil extracellular traps mediate COVID-19 pathology. J Exp Med.

[85] Radermecker C, Detrembleur N, Guiot J (2020). Neutrophil extracellular traps infiltrate the lung airway, interstitial, and vascular compartments in severe COVID-19. J Exp Med.

[86] Masso-Silva JA, Moshensky A, Lam MTY (2022). Increased peripheral blood neutrophil activation phenotypes and neutrophil extracellular trap formation in critically ill coronavirus disease 2019 (COVID-19) patients: a case series and review of the literature. Clin Infect Dis.

[87] Morrissey SM, Geller AE, Hu X (2021). A specific low-density neutrophil population correlates with hypercoagulation and disease severity in hospitalized COVID-19 patients. JCI Insight.

[88] Carissimo G, Xu W, Kwok I (2020). Whole blood immunophenotyping uncovers immature neutrophil-to-VD2 T-cell ratio as an early marker for severe COVID-19. Nat Commun.

[89] Parackova Z, Bloomfield M, Klocperk A, Sediva A (2021). Neutrophils mediate Th17 promotion in COVID-19 patients. J Leukoc Biol.

[90] Torres-Ruiz J, Absalón-Aguilar A, Nuñez-Aguirre M (2021). Neutrophil extracellular traps contribute to COVID-19 hyperinflammation and humoral autoimmunity. Cells.

[91] Schulte-Schrepping J, Reusch N, Paclik D (2020). Severe COVID-19 is marked by a dysregulated myeloid cell compartment. Cell.

[92] Hacbarth E, Kajdacsy-Balla A (1986). Low density neutrophils in patients with systemic lupus erythematosus, rheumatoid arthritis, and acute rheumatic fever. Arthritis Rheum.

[93] Denny MF, Yalavarthi S, Zhao W (2010). A distinct subset of proinflammatory neutrophils isolated from patients with systemic lupus erythematosus induces vascular damage and synthesizes type I IFNs. J Immunol.

[94] Midgley A, Beresford MW (2016). Increased expression of low density granulocytes in juvenile-onset systemic lupus erythematosus patients correlates with disease activity. Lupus.

[95] Keskinov AA, Shurin MR (2015). Myeloid regulatory cells in tumor spreading and metastasis. Immunobiology.

[96] Shaul ME, Eyal O, Guglietta S (2020). Circulating neutrophil subsets in advanced lung cancer patients exhibit unique immune signature and relate to prognosis. FASEB J.

[97] Fridlender ZG, Sun J, Kim S (2010). Polarization of TAN phenotype by TGFb: “N1” versus “N2” TAN. Cancer Cell.

[98] Zou J-M, Qin J, Li Y-C (2017). IL-35 induces N2 phenotype of neutrophils to promote tumor growth. Oncotarget.

[99] Ogura K, Sato-Matsushita M, Yamamoto S (2018). NK cells control tumor-promoting function of neutrophils in mice. Cancer Immunol Res.

[100] Massena S, Christoffersson G, Vågesjö E, Seignez C, Gustafsson K, Binet F, Herrera Hidalgo C, Giraud A, Lomei J, Weström S, Shibuya M (2015). Identification and characterization of VEGF-A–responsive neutrophils expressing CD49d, VEGFR1, and CXCR4 in mice and humans. Blood.

[101] Fridlender ZG, Sun J, Mishalian I (2012). Transcriptomic analysis comparing tumor-associated neutrophils with granulocytic myeloid-derived suppressor cells and normal neutrophils. PLoS ONE.

[102] Wang TT, Zhao YL, Peng LS (2017). Tumour-activated neutrophils in gastric cancer foster immune suppression and disease progression through GM-CSF-PD-L1 pathway. Gut.

[103] Cheng Y, Li H, Deng Y (2018). Cancer-associated fibroblasts induce PDL1+ neutrophils through the IL6-STAT3 pathway that foster immune suppression in hepatocellular carcinoma. Cell Death Dis.

[104] Huh SJ, Liang S, Liang S, Sharma A (2010). Transiently entrapped circulating tumor cells interact with neutrophils to facilitate lung metastasis development. Cancer Res.

[105] Eruslanov EB, Bhojnagarwala PS, Quatromoni JG (2014). Tumor-associated neutrophils stimulate T cell responses in early-stage human lung cancer. J Clin Investig.

[106] Wu P, Wu D, Ni C (2014). γδT17 cells promote the accumulation and expansion of myeloid-derived suppressor cells in human colorectal cancer. Immunity.

[107] Brandau S, Trellakis S, Bruderek K (2011). Myeloid-derived suppressor cells in the peripheral blood of cancer patients contain a subset of immature neutrophils with impaired migratory properties. J Leukoc Biol.

[108] Sreeramkumar V, Adrover JM, Ballesteros I (2014). Neutrophils scan for activated platelets to initiate inflammation. Science.

[109] Wang X, Qiu L, Li Z (2018). Understanding the multifaceted role of neutrophils in cancer and autoimmune diseases. Front Immunol.

[110] Armstrong DJ, Crockard AD, Wisdom BG (2006). Accelerated apoptosis in SLE neutrophils cultured with anti-dsDNA antibody isolated from SLE patient serum: a pilot study. Rheumatol Int.

[111] Dominical VM, Bértolo MB, Almeida CB (2011). Neutrophils of rheumatoid arthritis patients on anti-TNF-α therapy and in disease remission present reduced adhesive functions in association with decreased circulating neutrophil-attractant chemokine levels. Scand J Immunol.

[112] Rarok AA, Stegeman CA, Limburg PC, Kallenberg CGM (2002). Neutrophil membrane expression of proteinase 3 (PR3) is related to relapse in PR3-ANCA-associated vasculitis. J Am Soc Nephrol.

[113] Chakravarti A, Raquil M-A, Tessier P, Poubelle PE (2009). Surface RANKL of Toll-like receptor 4-stimulated human neutrophils activates osteoclastic bone resorption. Blood.

[114] Assi LK, See HW, Ludwig A (2007). Tumor necrosis factor α activates release of B lymphocyte stimulator by neutrophils infiltrating the rheumatoid joint. Arthritis Rheum.

[115] Nagase H, Miyamasu M, Yamaguchi M (2002). Cytokine-mediated regulation of CXCR4 expression in human neutrophils. J Leukoc Biol.

[116] Sachs UJH, Andrei-Selmer CL, Maniar A (2007). The neutrophil-specific antigen CD177 is a counter-receptor for platelet endothelial cell adhesion molecule-1 (CD31). JBC.

[117] de Bondt M, Hellings N, Opdenakker G, Struyf S (2020). Neutrophils: Underestimated players in the pathogenesis of multiple sclerosis (ms). Int J Mol Sci.

[118] Whittaker Hawkins RF, Patenaude A, Dumas A (2017). ICAM1+ neutrophils promote chronic inflammation via ASPRV1 in B cell–dependent autoimmune encephalomyelitis. JCI Insight.

[119] Naegele M, Tillack K, Reinhardt S (2012). Neutrophils in multiple sclerosis are characterized by a primed phenotype. J Neuroimmunol.

[120] Németh T, Sperandio M, Mócsai A (2020). Neutrophils as emerging therapeutic targets. Nat Rev Drug Discov.

[121] Dong Y, Lagarde J, Xicota L (2018). Neutrophil hyperactivation correlates with Alzheimer’s disease progression. Ann Neurol.

[122] Woodberry T, Bouffler SE, Wilson AS (2018). The emerging role of neutrophil granulocytes in multiple sclerosis. J Clin Med.

[123] Safi R, Kallas R, Bardawil T (2018). Neutrophils contribute to vasculitis by increased release of neutrophil extracellular traps in Behçet’s disease. J Dermatol Sci.

[124] Chiang C-C, Cheng W-J, Korinek M (2019). Neutrophils in psoriasis. Front Immunol.

[125] Talukdar S, Oh DY, Bandyopadhyay G (2012). Neutrophils mediate insulin resistance in mice fed a high-fat diet through secreted elastase. Nat Med.

[126] Brotfain E, Hadad N, Shapira Y (2015). Neutrophil functions in morbidly obese subjects. Clin Exp Immunol.

[127] Honda M, Kubes P (2018). Neutrophils and neutrophil extracellular traps in the liver and gastrointestinal system. Nat Rev Gastroenterol Hepatol.

[128] Wu X, Kim D, Young AT, Haynes CL (2014). Exploring inflammatory disease drug effects on neutrophil function. Analyst.

[129] Allegra L, Dal Sasso M, Bovio C (2002). Human neutrophil oxidative bursts and their in vitro modulation by different N-Acetylcysteine concentrations. Arzneimittel-Forschung/Drug Res.

[130] Palmér R, Mäenpää J, Jauhiainen A (2018). Dipeptidyl peptidase 1 inhibitor AZD7986 induces a sustained, exposure-dependent reduction in neutrophil elastase activity in healthy subjects. Clin Pharmacol Ther.

[131] Ali RA, Gandhi AA, Meng H (2019). Adenosine receptor agonism protects against NETosis and thrombosis in antiphospholipid syndrome. Nat Commun.

[132] Kubota S, Watanabe M, Shirato M (2015). An inhaled phosphodiesterase 4 inhibitor E6005 suppresses pulmonary inflammation in mice. Eur J Pharmacol.

[133] Aikawa N, Kawasaki Y (2014). Clinical utility of the neutrophil elastase inhibitor sivelestat for the treatment of acute respiratory distress syndrome. Ther Clin Risk Manag.

[134] Lipp P, Ruhnau J, Lange A, Vogelgesang A, Dressel A, Heckmann M (2017). Less neutrophil extracellular trap formation in term newborns than in adults. Neonatology S Karger AG.

[135] Yost CC, Schwertz H, Cody MJ, Wallace JA, Campbell RA, Vieira-De-Abreu A (2016). Neonatal NET-inhibitory factor and related peptides inhibit neutrophil extracellular trap formation. J Clin Investig.

[136] Denorme F, Portier I, Rustad JL, Cody MJ, de Araujo CV, Hoki C (2022). Neutrophil extracellular traps regulate ischemic stroke brain injury. J Clin Invest.

[137] Shishikura K, Horiuchi T, Sakata N, Trinh DA, Shirakawa R, Kimura T (2016). Prostaglandin E2 inhibits neutrophil extracellular trap formation through production of cyclic AMP. Br J Pharmacol.

[138] Tilgner J, Von Trotha KT, Gombert A, Jacobs MJ, Drechsler M, Döring Y (2016). Aspirin, but Not Tirofiban displays protective effects in endotoxin induced lung injury. PLoS ONE.

[139] Li M, Lin C, Deng H, Strnad J, Bernabei L, Vogl DT (2020). A novel peptidylarginine deiminase 4 (PAD4) inhibitor BMS-P5 blocks formation of neutrophil extracellular traps and delays progression of multiple myeloma. Mol Cancer Ther.

[140] Van Der Giessen LJ, Gosselink R, Hop WCJ, Tiddens HAWM (2007). Recombinant human DNase nebulisation in children with cystic fibrosis: before bedtime or after waking up?. Eur Respir J.

[141] Fuchs HJ, Borowitz DS, Christiansen DH, Morris EM, Nash ML, Ramsey BW (1994). Effect of aerosolized recombinant human dnase on exacerbations of respiratory symptoms and on pulmonary function in patients with cystic fibrosis. N Engl J Med.

[142] Silva CMS, Wanderley CWS, Veras FP, Gonçalves AV, Lima MHF, Toller-Kawahisa JE (2022). Gasdermin-D activation by SARS-CoV-2 triggers NET and mediate COVID-19 immunopathology. Crit Care.

[143] Wang Z, Cho J, Malik AB (2014). Prevention of vascular inflammation by nanoparticle targeting of adherent neutrophils. Nat Nanotechnol.

[144] Bachmaier K, Stuart A, Singh A (2022). Albumin nanoparticle endocytosing subset of neutrophils for precision therapeutic targeting of inflammatory tissue injury. ACS Nano.

[145] Cruz MA, Bohinc D, Andraska EA (2022). Nanomedicine platform for targeting activated neutrophils and neutrophil–platelet complexes using an α1-antitrypsin-derived peptide motif. Nat Nanotechnol.

[146] Khan MA, Palaniyar N (2017). Transcriptional firing helps to drive NETosis. Sci Rep.

